# Epidemiological characteristics and importance of lobulation of giant epidermal cysts: An 18-year retrospective review of 19 cases

**DOI:** 10.1097/MD.0000000000029978

**Published:** 2022-08-05

**Authors:** Kyu-Il Lee, Sik Namgoong, Hi-Jin You, Tae-Sung Jeon

**Affiliations:** a Department of Plastic Surgery, Korea University College of Medicine, Seoul, Republic of Korea; b Department of Pathology, Korea University College of Medicine, Seoul, Republic of Korea.

**Keywords:** cysts, epidemiology, epidermal cyst, neoplasms, tumor burden

## Abstract

Giant epidermal cysts, which have a diameter of ≥5 cm, have rarely been reported. Giant epidermal cysts that have multiple lobules are referred to as multilocular giant epidermal cysts. This study aims to establish the epidemiological characteristics and statistically determine the significance of lobulation in giant epidermal cysts.

Data on 19 patients who developed giant epidermal cysts between January 2003 and February 2021 were retrospectively reviewed. Patients were divided into 2 groups based on the presence of septa and the differences in characteristics were analyzed.

Among the 19 patients, 16 (84.2%) were male, and the mean age was 57.7 ± 10.6 years. The mean patient-reported tumor duration was 14.8 ± 12.5 years. Seven (36.8%) patients had multilocular giant epidermal cysts, whereas 12 (63.2%) had unilocular giant epidermal cysts. Compared with unilocular giant epidermal cysts, multilocular giant epidermal cysts had a significantly larger mean diameter (6.0 ± 0.7 vs 8.2 ± 1.8 cm, *P* = .02) and estimated volume (91.8 ± 43.3 vs 250.0 ± 157.0 mL, *P* = .02).

Giant epidermal cysts have distinctive epidemiologic characteristics with predominance among males, those in their 50s, and a long tumor duration. Multilocular giant epidermal cysts are significantly larger in diameter and volume than unilocular ones.

## 1. Introduction

Epidermal cysts are common cutaneous tumors filled with keratin debris.^[1-4]^ They are usually asymptomatic and have a predilection for hair-bearing areas of the body, such as the face, neck, scalp, and trunk.^[3,5]^ Most cysts grow slowly and are more susceptible to infection than other benign tumors, such as lipomas.^[1,5,6]^ Therefore, epidermal cysts are generally excised at an early stage or ruptured before growth. However, giant epidermal cysts (GECs), which have a diameter of ≥5 cm, have rarely been reported.^[2,5,7-10]^

Because of the rarity of these diseases, most GECs are reported as case reports. Among the published articles, the study with the largest number of cases included only 14 cases and focused only on the imaging characteristics.^[10]^ For a clinician to accurately manage the disease, knowledge of the epidemiological features of the disease as well as the imaging characteristics is necessary. However, as GECs are rare, studies on their epidemiology have not been performed.

**Figure 1.**
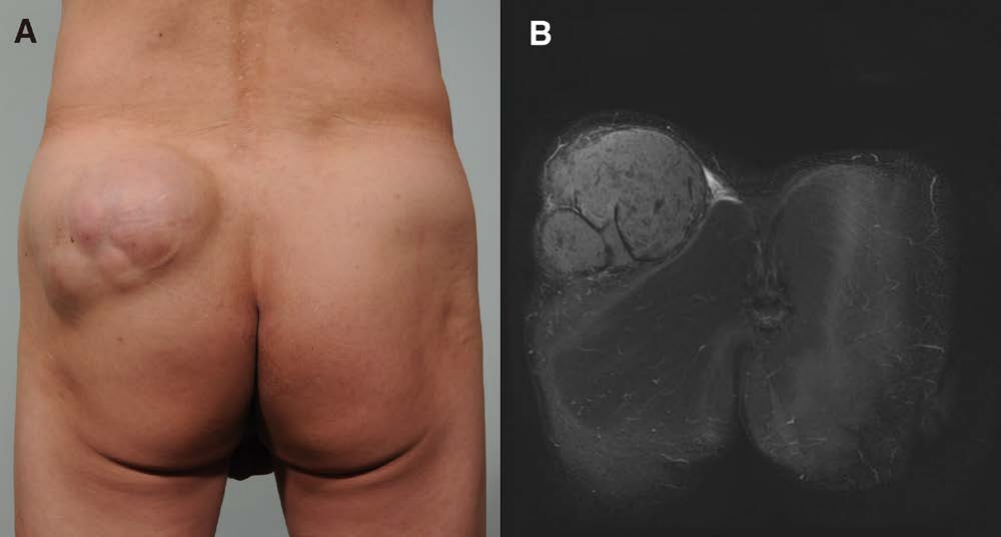
(A) Case 7: The largest MGEC in this study, located on the left buttock (10 × 7 × 6 cm^3^). Multiple lobules are observed on the surface. (B) MR images demonstrating hyperintense STIR signal intensity lesion with multiple lobules. MGEC = multilocular giant epidermal cyst, STIR = short tau inversion recovery.

Unlike conventional epidermal cysts, GECs often have internal septa. Since the first report by Fujiwara et al, GECs with multiple lobules have been termed multilocular GECs (MGECs) (Fig. 1). In their study, they reported that MGECs are more common in older men and in those with thick skins and skins uncared for a long period.^[8,9]^ However, it is unclear whether these features are of MGECs or unilocular GECs (UGECs) as statistical comparisons between MGECs and UGECs have not been performed. Thus, the significance of lobulation in GECs has not yet been investigated.

In this study, we examined 19 cases of patients diagnosed with GECs who underwent surgery at our institution within the past 18 years. This study aimed to establish the epidemiological characteristics and statistically determine the significance of lobulations in GECs. To the best of our knowledge, this study has the largest number of cases of GECs to date.

## 2. Methods

### 2.1. Study subjects

The study protocol was approved by the institutional review board (IRB) of Korea University Guro Hospital (IRB No: 2020AS0354). The study was performed in full compliance with the principles of the Declaration of Helsinki, and all patients provided informed consent.

This retrospective study reviewed patients with epidermal cysts at our institution between January 2003 and February 2021. Based on the pathology reports and imaging tests, patients with GECs were included in our study. Patients were excluded if any of the following were present: insufficient medical records, without preoperative photos, and lesion size <5 cm. Finally, a total of 19 patients (12 and 7 with UGECs and MGECs, respectively) were enrolled.

Patients’ baseline demographics, imaging test findings, postoperative complications, and length of drain tube (in days) were reviewed. Preoperative digital images were used to determine the presence of puncta, lesion locations, and the affected side.

### 2.2. Size and volume measurement

Internal keratin debris can spill out during excisional biopsy; hence, the longest diameter of the lesions were measured based on imaging tests, such as CT and MRI. However, when only ultrasonography was performed or imaging results were unavailable, the size and lobulation were determined based on the tissue specimen. The estimated volume, which must be higher than the actual volume, was calculated as the maximum cross-sectional area of the cyst multiplied by the height perpendicular to its area. As the GECs have irregular surfaces, approximated values were used. Additionally, to reduce investigator bias, each investigator measured the lesions thrice, and the mean values were recorded.

### 2.3. Intergroup comparison

To determine the significance of lobulations in GECs, the patients were classified as having UGECs or MGECs. After collecting information on sex, age, lesion diameter and volume, disease duration, and the affected side, statistical analyses were performed.

Data were collected using Microsoft Excel (Microsoft Corp., Redmond, WA) and analyzed using IBS SPSS (version 23.0; IBM Corp., Armonk, NY). Continuous and categorical variables of the UGEC and MGEC groups were compared using the Mann-Whitney *U* test and Fisher exact test, respectively. All *P* values were 2-sided, and statistical significance was set at *P* < .05.

## 3. Results

**Figure 2.**
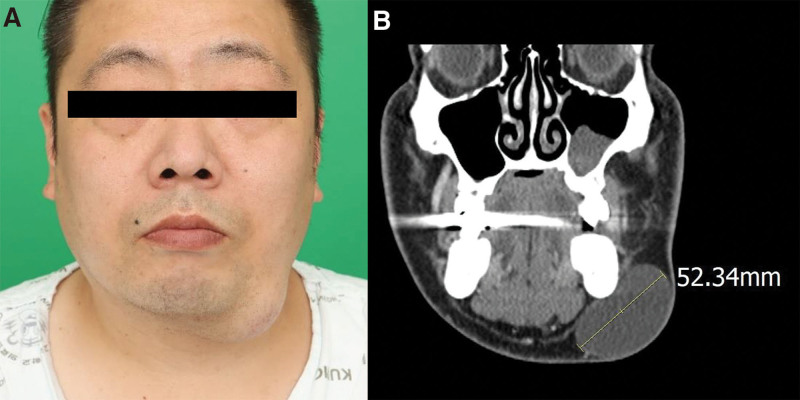
(A) Case 10: A rare case of UGEC located on the left chin. (B) Coronal computed tomography showing a 5.2 × 4.3 × 2.8 cm^3^ UGEC. UGEC = unilocular giant epidermal cyst.

A total of 19 GECs were surgically treated at our institution between January 2003 and February 2021. The baseline demographic and clinical features of the GECs are listed in Table 1. Among the 19 patients, 16 (84.2%) were male, and the mean age was 57.7 ± 10.6 years. The mean patient-reported tumor duration was 14.8 ± 12.5 years. Seven (36.8%) patients had MGECs, whereas 12 (63.2%) had UGECs. The mean lesion diameter and estimated volume were 6.8 ± 1.6 cm and 150.1 ± 124.6 cm³, respectively. Regarding the affected regions of the body, 17 (89.5%) patients had posterior-sided GECs, while 2 (10.5%) had anterior-sided GECs. The anterior locations were on the chin and abdomen (Fig. 2).

**Table 1:** Baseline demographic characteristics of patients with unilocular GECs and multilocular GECs†.

Variables	All patients (N = 19)	Unilocular (N = 12)	Multilocular (N = 7)	*P* ‡
Sex, n (%)				1.000
Male	16 (84.2%)	10 (83.3%)	6 (85.7%)	
Female	3 (15.8%)	2 (16.7%)	1 (14.3%)	
Age (y)	57.7 ± 10.6	57.3 ± 11.6	58.4 ± 9.5	.967
Greatest diameter (cm)	6.8 ± 1.6	6.0 ± 0.7	8.2 ± 1.8	.017*
Estimated volume (cm³)	150.1 ± 124.6	91.8 ± 43.3	250.0 ± 157.0	.022*
Duration (y)	14.8 ± 12.5	17.2 ± 11.9	10.7 ± 13.4	.142
Affected side, n (%)				.509
Anterior	2 (10.5%)	2 (16.7%)	0 (0%)	
Posterior	17 (89.5%)	10 (83.3%)	7 (100%)	

The results of the comparison between UGECs and MGECs are also summarized. Compared to UGECs, MGECs had a significantly larger mean diameter (6.0 ± 0.7 vs 8.2 ± 1.8 cm, *P* = .02) and estimated volume (91.8 ± 43.3 vs 250.0 ± 157.0 mL, *P* = .02). Overall, in both groups, older men were predominantly affected and primarily had posterior-side lesions. No significant differences were observed between the groups in terms of sex ratio, age, tumor duration, and the affected side.

Detailed clinical characteristics are listed in Table 1, Supplemental Digital Content, http://links.lww.com/MD/G963. Puncta, which enable a patient to recognize the lesion, were observed in only 1 patient (case 15). Regarding the preoperative imaging workup, only 2 patients underwent no imaging tests. Drainage was routinely performed to prevent fluid collection, except in 2 patients with relatively small volumes (cases 8 and 10). In these patients, compression dressing with elastic bandages was performed.

Three patients had postoperative complications as follows: 1 (case 12) developed hematoma on postoperative day 1 and underwent emergency hematoma evacuation under local anesthesia; 1 (case 18) with seroma formation underwent serial percutaneous syringe aspiration in an outpatient clinic; and 1 (case 5) experienced paresthesia at the surgical site, which resolved spontaneously after 3 months. All complications were resolved with proper management.

## 4. Discussion

This study revealed that GECs were prevalent in men (sex ratio, 5.3:1) and in those in their 50s. GECs were mostly found on the posterior side of the body, had a long tumor duration, and had almost no punctum. These findings differ from the epidemiology of typical epidermal cysts (predominantly male [sex ratio, 2:1]; usually occurring in the third and fourth decades of life; typically involving the face, neck, preauricular area, or upper trunk; and usually presenting with a punctum).^[1]^

A possible explanation for the sex ratio is that men have thicker skin on which cysts can grow for a long period without rupturing^[11]^ and GECs are mainly found in the thick-skinned areas of the body, such as the buttock, back, posterior scalp, and posterior neck.^[12]^

In addition, GECs were mainly posterior-sided where they could be easily covered by hair or clothing and become relatively inconspicuous; therefore, patients may have hesitated to undergo surgery. Furthermore, the absence of a punctum might have delayed disease detection.^[8, 9]^ Therefore, because GECs were left unattended for a long period without rupture, their age at onset was approximately 10 years higher than that of usual epidermal cysts.

Another consideration is the significance of lobulation in GECs. Fujiwara et al analyzed 8 consecutive cases of MGECs and reported that MGECs had clinical features that distinguished them from ordinary epidermal cysts, such as a male predominance, long disease duration, absence of punctum, and thick dermal layer locations.^[8,9]^ However, because of the relatively small number of cases, it is unclear whether these characteristics are those of MGECs or GECs. We analyzed more cases and found that MGECs had a larger lesion size and volume than UGECs, but other epidemiological characteristics were not significantly different. Therefore, the characteristics mentioned by Fujiwara et al should be regarded as those of GECs in general.

**Figure 3.**
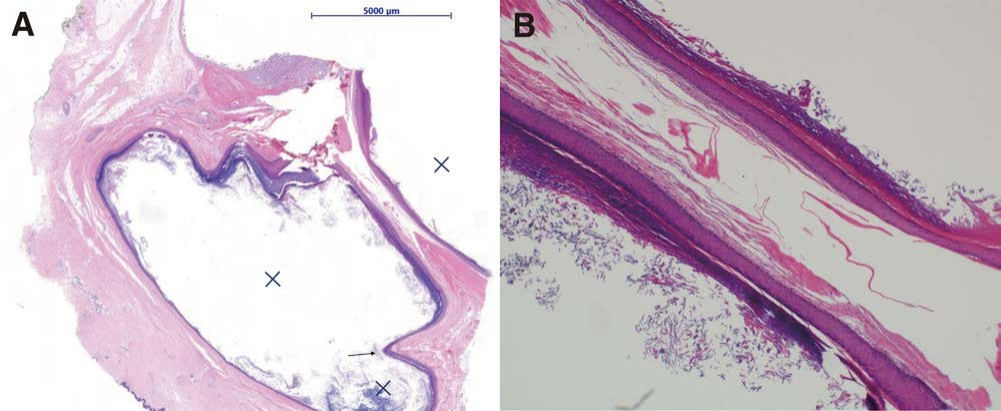
(A) Histological examination of the MGEC shows the septum that divides the cyst. The lobules are marked with X and an incomplete septum is observed (arrow) (hematoxylin-eosin stain; scale bar, 5000 µm). (B) The septa of MGEC are lined with keratinized stratified squamous epithelium (hematoxylin-eosin stain; 40× magnification). MGEC = multilocular giant epidermal cyst.

Histological examination provides reasonable inference regarding the difference in the sizes of UGECs and MGECs (Fig. 3). Microscopically, septa are mostly incomplete; therefore, the lobes communicate with each other. A possible hypothesis is that these septa are formed as the surrounding thick dermal layer is pushed out while the cysts grow. Presumably, epidermal cysts do not merge to form MGECs. Instead, a single epidermal cyst grows and divides into several lobes.^[2,8,9]^ Although the mass enlarges, the area that fails to adapt and grow becomes the septa. However, other factors should be considered because there are cases of small epidermal cysts with multiple lobules.

**Figure 4.**
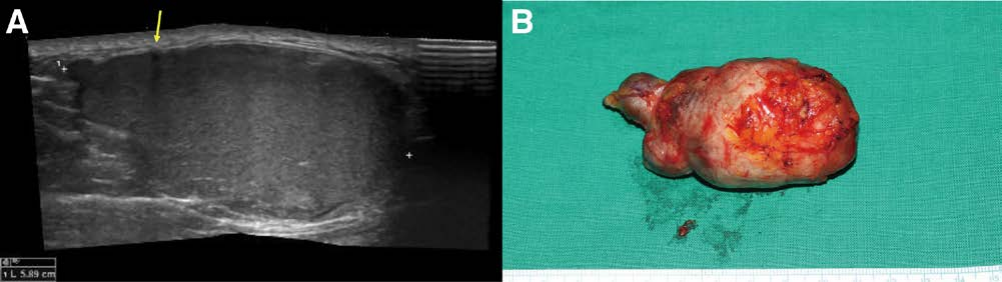
(A) Case 1: Ultrasonography shows a hypoechoic linear line indicating a septum (arrow) inside the hyperechoic mass with keratin debris. (B) The photo of the specimen shows a 6.8 × 3.5 × 3.5 cm^3^ multilocular GEC similar to the ultrasonographic findings. GEC = giant epidermal cyst.

Masses that exhibit recent growth or have a lesion diameter of ≥5 cm have a 20% likelihood of being malignant.^[10]^ Therefore, for presumed GECs, preoperative imaging examinations, such as ultrasonography, CT, and MRI, should be conducted to exclude malignancy and other benign soft tissue tumors. Preoperative evaluation using ultrasound as well as CT or MRI could also be usefully performed to predict MGECs. The septa were predicted through the hypoechoic linear line inside the mass, which had a high degree of similarity to the postoperative specimen (Fig. 4). However, in our study, 2 patients did not undergo preoperative imaging as 1 had a clearly visible punctum and the other refused the tests due to cost.

In our patients, postoperative complications included seroma, hematoma, and temporary paresthesia. No correlation was found between the incidence of postoperative complications and the size of the lesion. The tendency of fluid collection may be affected by the location rather than size of the lesion. Patients with seroma or hematoma developed lesions on relatively mobile areas, such as the buttocks and scapula. Therefore, after thorough intraoperative hemostasis, the surgical site should be immobilized and compressed for 4 to 5 days after drain insertion. Meanwhile, the dissection of epidermal cysts from surrounding tissues is relatively easy, and therefore, permanent nerve damage or functional dysfunction is rare.

This study has limitations. First of all, due to the retrospective nature of our study, some of our data were inaccurate or unavailable. For example, patients without preoperative clinical photos or baseline demographic data were excluded from the study. In addition, the disease duration might be inaccurate as it was self-reported. Second, lesions originating from the sole, a possible site for GECs, were not found because of the small number of cases.^[2,7,9]^ Similarly, no cases of malignant transformation to squamous cell carcinoma were identified.^[13-19]^ Finally, due to the rarity and long disease period of the disease, we could not elucidate an evident mechanism for the size difference between UGEC and MGECs. Instead, we performed clinical inferences through histological examination.

Despite these limitations, our study has the following strengths. To our knowledge, our study included a larger number of GEC patients than any previous studies. Among those rare cases, there was even a patient with a maximum lesion diameter of 10 cm. Furthermore, our study statistically determined for the first time that MGECs were larger in size than UGECs. Finally, we also found that what had been presumed to be epidemiological features of MGECs in previous studies could be attributed to GECs.

In conclusion, GECs exhibit distinctive characteristics of male predominance and have a longer tumor duration than typical epidermal cysts. They are mainly located on relatively thick dermal layers and in areas that can be easily hidden by hair or clothing. Furthermore, MGECs are significantly larger in diameter and volume than UGECs. A true mechanism for their size differences is still unknown. However, clinical inferences can be performed through histologic examination. In histological analysis, MGEC did not appear to arise from the fusion of multiple individual epidermal cysts. In contrast, considering that incomplete septa are the majority in histological analysis, it can be inferred that septation occurs during the growing process of squamous epithelial cells pushing out the limited space outside. With some limitations, further research with larger cases, prospective nature, and more evident pathologic analysis are needed.Author contributionsKyu-Il Lee: Performed the study and wrote the draftSik Namgoong: Performed the study and edited the draftHi-Jin You: Performed the study and analyzed the clinical dataTae-Sung Jeon: Analyzed the pathologic data

## Supplementary Material



## Footnotes

* *P* < .05.

† Data are presented as number (percentage) or mean ± standard deviations.

‡ *P* values were calculated using the Mann-Whitney *U* test for continuous variables and the Fisher exact test for categorical variables.
