# Supplementary material for: Epidemiological characteristics and importance of lobulation of giant epidermal cysts: An 18-year retrospective review of 19 cases
Source: Medicine (Baltimore). 2022 Aug 5;101(31):e29978. doi: 10.1097/MD.0000000000029978 (PMC9351876; doi:10.1097/MD.0000000000029978)
Supplement: Supplementary file 1 [file medi-101-e29978-s001.pdf]

Supplementary Table 1. Cases of giant epidermal cysts

Case	Sex	Age (y)	Lobulation	Duration (y)	Lesion location	Affected side	Greatest diameter (cm)	Estimated volume (cm³)	Punctum	Imaging test	Drain	Postoperative Complications
1	M	60	Multilocular	1	Posterior thigh	P	5	83.3	-	US	O	-
2	M	46	Multilocular	1	Buttock	P	6.8	83.3	-	US	O	-
3	F	52	Multilocular	30	Posterior scalp	P	9	126	-	CT	O	-
4	M	50	Multilocular	5	Posterior axilla	P	9.5	257	-	CT	O	-
5	M	73	Multilocular	30	Posterior scalp	P	8	384	-	MRI	O	Paresthesia
6	M	64	Multilocular	1	Flank	P	10	413	-	CT	O	-
7	M	64	Multilocular	7	Buttock	P	10	420	-	MRI	O	-
8	F	67	Unilocular	15	Posterior leg	P	5.2	25.3	-	US	-	-
9	M	59	Unilocular	8	Abdomen	A	5.2	46.8	-	US	O	-
10	M	40	Unilocular	5	Anterior chin	A	5.2	62.6	-	CT	-	-
11	M	56	Unilocular	15	Posterior scalp	P	6.2	77.5	-	CT	O	-
12	M	63	Unilocular	3	Buttock	P	6.9	77.6	-	CT	O	Hematoma
13	M	54	Unilocular	30	Posterior neck	P	5.4	79.4	-	CT	O	-
14	M	62	Unilocular	10	Buttock	P	6.3	88.2	-	CT	O	-
15	F	36	Unilocular	20	Posterior thigh	P	6.7	93.8	O	None	O	-
16	M	79	Unilocular	45	Posterior scalp	P	5.6	98.6	-	CT, MRI	O	-
17	M	55	Unilocular	10	Posterior scalp	P	6.3	130	-	CT	O	-
18	M	65	Unilocular	25	Scapula	P	6	132	-	None	O	Seroma
19	M	52	Unilocular	20	Buttock	P	7.3	190	-	MRI	O	-

A, anterior; CT, computed tomography; F, female; M, male; MRI, magnetic resonance imaging; O, present; P, posterior; US, ultrasonography

Supplementary Table legend:

Supplementary Table 1. Cases of giant epidermal cysts

Abbreviations: A, anterior; CT, computed tomography; F, female; M, male; MRI, magnetic resonance imaging; O, present; P, posterior; US, ultrasonography
